# Identification of the Pelvic Organ Prolapse/Incontinence Sexual Questionnaire—IUGA Revised (PISQ-IR) Cutoff Scores for Impaired Sexual Function in Women with Pelvic Floor Disorders

**DOI:** 10.3390/jcm9010013

**Published:** 2019-12-19

**Authors:** Magdalena Emilia Grzybowska, Konrad Futyma, Dariusz Wydra

**Affiliations:** 1 Department of Gynecology, Gynecologic Oncology and Gynecologic Endocrinology, Medical University of Gdańsk, Smoluchowskiego 17, 80-214 Gdańsk, Poland; dwydra@icloud.com; 2 2nd Department of Gynecology, Medical University of Lublin, Jaczewskiego 8, 20-954 Lublin, Poland; futymakonrad@mp.pl

**Keywords:** pelvic floor disorders, quality of life, sexual function, cutoff value, Pelvic Organ Prolapse/Incontinence Sexual Questionnaire—IUGA Revised (PISQ-IR), sexual dysfunction, Female Sexual Function Index, sensitivity, specificity

## Abstract

The aim of this cross-sectional study was to determine the cutoff scores for sexual dysfunction in disease-specific Pelvic Organ Prolapse/Incontinence Sexual Questionnaire—IUGA Revised (PISQ-IR) for women with pelvic floor disorders (PFD). Medical history and urogynecological data of 521 women with PFD were collected. The subjects provided information about their sexual activity and completed Female Sexual Function Index (FSFI) and PISQ-IR questionnaires. Sexually active (SA) women were further analyzed and categorized using their FSFI scores: <26.55—sexual dysfunction, >26.55—no sexual dysfunction. Receiver operating characteristics (ROC) curve tested how well PISQ-IR allowed to discriminate between patients with and without sexual disorders. Area under curve (AUC) was calculated to measure the PISQ-IR Summary Score efficiency in the prediction. The cutoff values which minimalize (1-specifity) and maximize sensitivity were selected. In the analyzed cohort, 250 (48%) women were SA and a total of 226 SA were recruited for the study: 143 (63.3%) with <26.55 FSFI and 83 (36.7%) with >26.55 FSFI (response rate: 90.4%). Using ROC curve analysis, PISQ-IR Summary Score of 2.68 was determined to be the optimal cutoff for distinguishing between dysfunctional and nondysfunctional women (AUC = 0.85), allowing to diagnose sexual dysfunction in SA women with PFD, with 90% sensitivity and 71% specificity.

## 1. Introduction

Pelvic floor disorders (PFD) include urinary incontinence (UI), pelvic organ prolapse (POP), and fecal incontinence. One-fourth of adult women in the United States (U.S.) report at least one PFD, and the prevalence significantly increases with age, from 31.6% between the ages of 50–59, up to 52.7% among 80-year-olds and older [1]. In light of the fact that female life expectancy in 2016 was reported to be 80.8 years in Europe, 83.2 in the United Kingdom, and 81.0 in the U.S. (World Health Organization), PFD constitute a widespread public health burden and may have a considerable impact on the quality of patient life and functioning, not only in terms of daily activities, but also significantly deteriorated sexual function (SF) [2]. According to Li-Yun-Fong et al., the percentage of sexually active (SA) women among those with PFD was 59% [3]. PFD has been associated with decreased SF, including reduced sexual arousal, lower orgasm frequency, and dyspareunia [4].

Additionally, the impact of the PFD-related treatment cannot be overlooked. The incidence rate of prolapse surgery varied from 100.9 to 168.0 per 100,000 women in the U.S. in 2007 and in Denmark in 2009, respectively [5,6]. The lifetime risk of surgery for either stress UI or POP is 20.5% [7]. Nowadays, patient-reported outcomes are vital elements of the assessment of the surgical or conservative treatment. The anatomical success of the treatment is no longer enough and, in consequence, the subjective assessment takes on an increasingly important role. Surgical correction of POP and/or UI improves SF in approximately 70% of the patients, although some studies that rely on the use of non-condition-specific questionnaires as outcome measures indicate no change [8]. Depending on the center, a variety of procedures are performed to treat PFD. The surgical techniques use different approaches: vaginal, abdominal, or minimally invasive surgeries. Complication and recurrence rates after various procedures differ substantially, and a good outcome measure is necessary to prove benefits of particular methods.

Therefore, it is important to use the recommended, validated, and well-researched instruments to assess the quality of life before and after treatment. The assessment of SF is typically based on self-administered questionnaires, with the Female Sexual Function Index (FSFI) among the most popular tools [9]. It is used to assess the effects of both the surgical and the conservative treatment [10]. However, as a generic questionnaire, it does not include questions targeting symptoms typical for PFD. General questionnaires may not be sensitive enough to detect differences caused by PFD [8]. The disease-specific Pelvic Organ Prolapse/Incontinence Sexual Questionnaire—IUGA Revised (PISQ-IR) was introduced in 2013 [11] and its usage gradually increases [12]. Still, as a fairly new instrument, it has no fixed cutoff values. Calculation of the threshold for distinguishing sexual dysfunction would improve clinical utility of the questionnaire and add another important measurement feature. Therefore, the aim of the study was to find the PISQ-IR cutoff scores for impaired sexual function in women with PFD.

## 2. Materials and Methods

**Figure 1:**
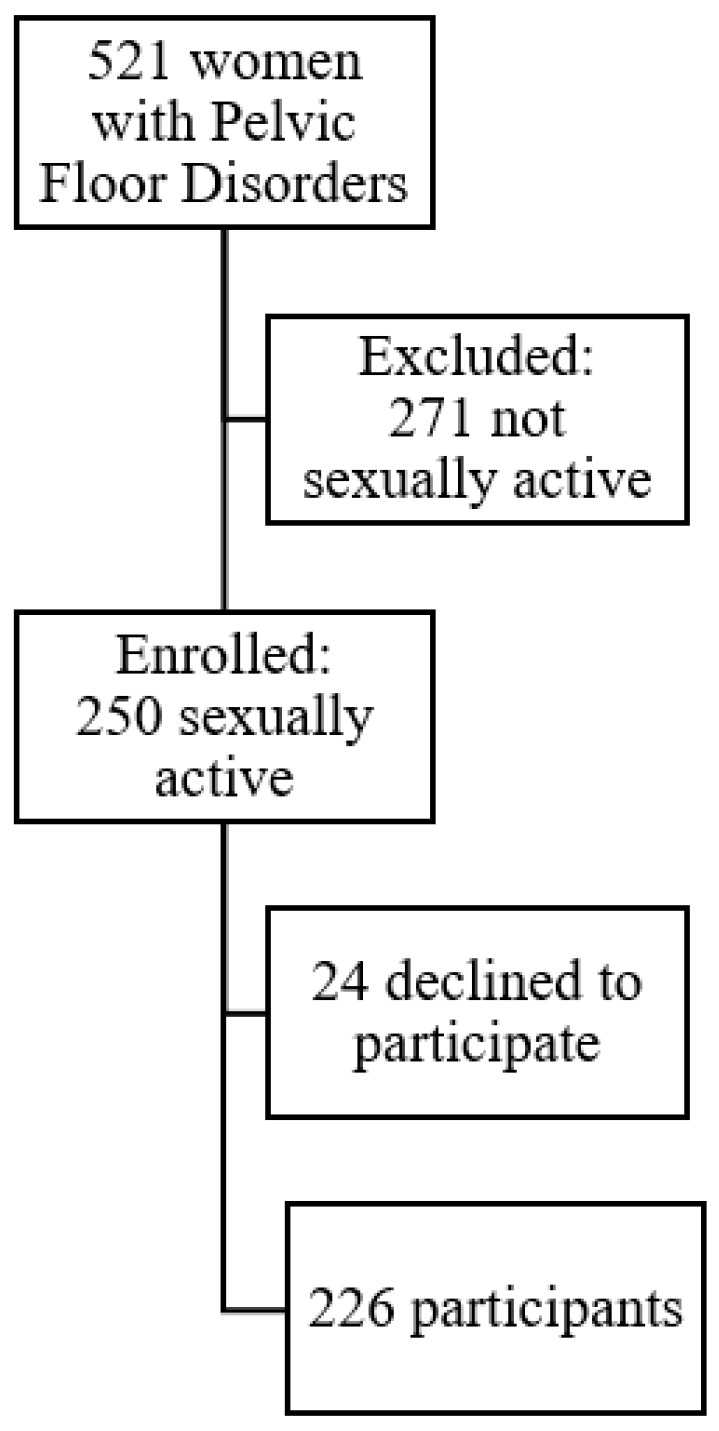
Study flow chart.

A cross-sectional study was conducted in 521 women referred to a gynecology clinic due to PFD complaints. Medical history was taken and urogynecological examination was performed according to the International Continence Society (ICS) standards; standardized terminology was used [13,14]. Patients were asked about their UI and fecal incontinence (FI) symptoms. Cough and Valsalva stress tests were conducted. The degree of prolapse was assessed using the Pelvic Organ Prolapse Quantification (POP-Q) system. Additionally, the subjects provided information on their sexual activity and completed two questionnaires: general (FSFI) and disease-specific (PISQ-IR). Sexually active women with PFD were enrolled for further analysis and categorized according to their FSFI scores: <26.55—sexual dysfunction and >26.55—no sexual dysfunction [9]. Medical history, urogynecological examinations, and questionnaire scores were compared. The enrollment process is shown in Figure 1.

### 2.1. The Applied Questionnaires

FSFI is a validated general questionnaire [15] widely used in clinical practice as a screening tool for female sexual dysfunction, as well as in clinical trials as an outcome measure [16]. It consists of 19 items, grouped into six domains: Desire, Arousal, Lubrication, Orgasm, Satisfaction, and Pain. The total score is obtained by adding the six domain scores (range: 2.0–36.0). Higher scores in particular domains and total FSFI score indicate better sexual function. The items in the questionnaire pertain to the last 4 weeks of activity. The cutoff score for differentiating women with and without sexual dysfunction is 26.55 (Table 1) [9].

PISQ-IR is a disease-specific questionnaire which was developed on the basis of the PISQ-12, to assess SF in both sexually active and inactive women with PFD [11,17]. It has been validated in ten languages so far [18,19]. PISQ-IR consists of two parts. Part 1, for not SA (NSA) women, where higher scores indicate a greater impact of the condition on sexual inactivity. Part 2, for SA women, with higher scores indicating better sexual function. The patient completes either six domain-specific subscales for SA women or four domain-specific subscales for NSA women. The domains can be scored using either mean calculation or transformed summation. Although either method is recommended to score the PISQ-IR domains, the most efficient way of calculating the Summary Score is achieved using a mean calculation (nonweighted) rather than a transformed sum (weighted scores) [20].

The Summary Score calculation for the SA scale should use a mean calculation by first summing the valid responses to items in the scale and then dividing the result by the number of items with valid responses. A valid response is provided for at least half the items of the SA scale. Items 11, 14a, 14b, 16, 17, 18, 19a, 19b, and 19c are reversed scored. Higher scores indicate better sexual function (Table 2) [20].

### 2.2. Statistical Analysis

Statistical analysis was performed with IBM SPSS Statistics 24.0 (SPSS Inc., Chicago, IL, USA).

All continuous variables are expressed as means and standard deviations. Categorical variables are expressed as percentages of the total group. A *p*-value of <0.05 was considered statistically significant, and all statistical tests were two-sided. ANOVA with Student’s t test was used for continuous variables, comparisons of categorical variables were performed using the Chi-square test and nonparametric Mann–Whitney U test. Receiver operating characteristics (ROC) curve was used to test how well PISQ-IR allowed to discriminate between patients with and without sexual disorders. AUC (area under curve) statistic was calculated to measure the PISQ-IR Summary Score efficiency in the aforementioned prediction. A ROC curve is a graphical illustration created by plotting the true-positive rate (sensitivity) against the false-positive rate (1-specificity), with AUC as statistic indicating the best cutoff point. The cutoff values which minimalize (1-specifity) and maximize sensitivity were selected. A subject-to-item ratio of 10:1 has been recommended for psychometric work, so a sample size of at least 120 SA women was required.

All subjects gave their informed consent for inclusion before they participated in the study. The study was conducted in accordance with the Declaration of Helsinki, and the protocol was approved by the Local Ethics Committee—Independent Bioethics Commission for Research, Medical University of Gdańsk, (No. NKBBN/219/2015, 26 May 2015).

## 3. Results

**Figure 2:**
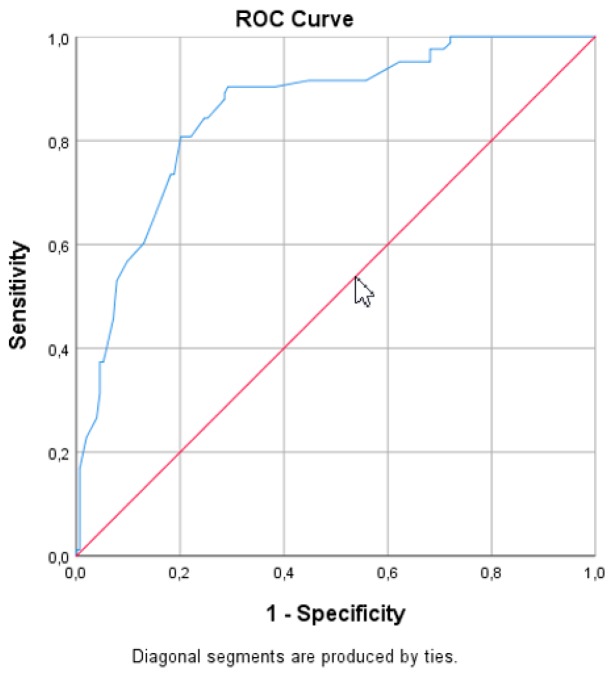
ROC (receiver operating characteristics) curve for sexual dysfunction for PISQ-IR.

Out of 521 women with PFD, 250 (48%) were SA and a total of 226 SA women were recruited for the study, with the response rate of 90.4%. Mean age of the study group was 56.6 ± 10.7, and mean body mass index (BMI) was 27.4 ± 4.4 kg/m^2^. In our study group, 53 (23.4%) subjects were diagnosed with UI, 88 (38.9%) with POP, 84 (37.2%) with both POP and UI, and 10.2% with fecal incontinence (FI). The POP-Q assessment revealed 8 (3.5%) women at stage 0, 14 (6.2%) at stage 1, 73 (32.3%) at stage 2, 110 (48.7%) at stage 3, and 21 (9.3%) at stage 4. After dichotomizing patients according to their FSFI results, we received two groups: 143 (63.3%) women with <26.55 FSFI and 83 (36.7%) with >26.55 FSFI. No differences were found between age, education, BMI, type of delivery, marital status, POP-Q stage, history of previous surgeries, and the diagnosis of various PFD between those groups. Women with FSFI scores indicating sexual dysfunction had higher parity and were more often postmenopausal (*p* = 0.02) (Table 3). Mean PISQ-IR Summary Score and Total FSFI score were 2.55 ± 0.38 and 20.65 ± 4.34 in the group with sexual dysfunctions, respectively and 3.06 ± 0.31 and 29.58 ± 2.01 in the group classified as nondysfunctional, respectively (Table 4). On the basis of the ROC curve analysis, the PISQ-IR Summary Score of 2.68 was determined to be the optimal cutoff for distinguishing between dysfunctional and nondysfunctional women (Figure 2). The assumption that women with scores above the cutoff values have good sexual function resulted in the true-positive rate of 90% (sensitivity), and represented the proportion of SA women who were correctly classified by the PISQ-IR Summary Score as “healthy”. The false-positive rate of 29% (1-specificity) represented the proportion of women with sexual dysfunction who were incorrectly classified as “healthy” (Figure 2).

For the statistical description of the effectiveness of classifying the subjects into two FSFI groups on the basis of PISQ-IR, an area under curve (AUC) of 0.85 was deemed the scale suitable for that purpose. The cutoff score of 2.68 for the PISQ-IR Summary Score allowed for the diagnosis of sexual dysfunction in SA women with PFD with 90% sensitivity and 71% specificity; 71% of the cases were correctly classified as dysfunctional, and 90% as nondysfunctional. Subjects with the PISQ-IR Summary Score of >2.68 were categorized as having “no sexual dysfunctions”, and those with the PISQ-IR Summary Score of <2.68 were recognized as having impaired sexual function.

## 4. Discussion

The assessment of the female sexual function is an important parameter of patient well-being. The current WHO definition of health encompasses its sexual aspect as an inseparable component of one’s perception of health. Both the baseline and post-treatment condition require reliable tools to assess sexual function of a patient. Even the Food and Drug Administration recommended the use of patient-reported outcomes for the evaluation of sexual function [13]. The PISQ-IR questionnaire was introduced already in 2013, but neither the cutoff scores nor the minimally important difference (MID) have been established since then. The reason for that might be the relatively complicated system of calculating the scores, which hinders a widespread use of the questionnaire, although the scoring program can be obtained from the IUGA website (https://www.iuga.org/resources/pisq-ir). PISQ-IR is one of the few condition-specific questionnaires recommended by the International Consultation of Incontinence (ICI). Other recommended tools include ICIQ-FLUTSsex (BFLUTS) (International Consultation on Continence Questionnaire—Female Lower Urinary Tract Symptoms Sex), ICIQ-VS (International Consultation of Incontinence Questionnaire—Vaginal Symptoms), SFQ (Sexual Function Questionnaire), and SQOL-F (Sexual Quality of Life—Female). The remaining two tools are the prototypes of the PISQ-IR, that is, the Pelvic Organ Prolapse Urinary Incontinence Sexual Questionnaire (PISQ) and its short version, the PISQ-12 [13]. Pelvic floor condition-specific measures are more likely to be responsive to change than measures which are not condition-specific. Additionally, some general quality of life questionnaires contain items pertaining to sexual function, for example the King’s Health Questionnaire [21].

According to ICI, three grades of recommendation (A, B, C) may be applied to the questionnaires. A “grade C” questionnaire is considered as “potential” and its published data confirm its validity or reliability or responsiveness to change, “grade B” is “recommended” (valid and reliable), and “grade A” is “highly recommended” (valid and reliable and responsive to change) [22]. According to the recent issue of the ICS Standards 2019, the PISQ-IR and PISQ-12 questionnaires are listed as “not rated” and PISQ as “grade B” [13]. However, in the previous ICS publication, PISQ-12 was graded as “B”, and PISQ-IR as “C” or even as “A” for the evaluation of quality of life in women with POP [22]. In light of such conflicting and confusing reports, those tools need to be better studied, not only in terms of validity and reliability, but also responsiveness to change. Defining the cutoff scores, which so far have not been established for the tools in question, constitutes yet another step in learning the properties of the questionnaire. MID, with the value of 6 points, was established only for PISQ [23].

The FSFI, although being a general questionnaire, has a “grade A” recommendation by the ICI, as an instrument which assesses multiple dimensions of sexual function [13], so it was selected as the comparative tool to determine the PISQ-IR cutoff values. FSFI has high feasibility and is used to assess the effectiveness of the treatment and how it impacts sexual function [2,10,12]. FSFI is commonly used, probably due to its generalizability and a user-friendly, uncomplicated scoring system. However, a recent systematic review by Neijenhuijs et al. has revealed some missing information concerning the psychometric properties of the instrument, its strengths notwithstanding [16]. According to these authors, the literature offers no data on the measurement error, responsiveness, and cross-cultural validity of FSFI. What could be controversial, they also stated that it is not possible to ensure that the test would be interpreted similarly in different cultures and no measure of systematic and random error in the change scores was reported [16].

Nonetheless, the applied FSFI cutoff score of 26.55 [9] allowed to classify 63.3% of the SA women from our study into the group with sexual dysfunction. In a study by Salonia et al., the percentage of women with sexual dysfunction among PFD subjects was 46%, however their study group was younger—mean age 52 (range 19–66), with the diagnosis of UI and lower urinary tract symptoms (LUTS) (i.e., dysuria, urgency-frequency syndrome, and recurrent bacterial cystitis) and no information on POP was provided [2]. POP-related bother has been reported to significantly influence self-perceived body image and sexual function [24]. Our study group comprised women with all types of PFDs, including UI, POP, and FI, which is a definite strength of our study. The patients were recruited from a university-based clinic, where they were referred for medical help. This could explain the higher percentage of women with FSFI scores indicating sexual dysfunction (63.3%). According to the literature, increased level of symptom bother is the main factor for seeking medical help among women with PFD [25]. Tinetti et al. reported sexual dysfunction in as many as 77.9% of women with PFD aged over 55 years [25].

The distinctive features of PISQ-IR include questions for sexually inactive women, who can additionally indicate reasons behind this. It allows also to analyze patients who are SA but without a partner and incorporates gender-neutral questions (item #11 and #12), which is in line with the current recommendations [13]. The PISQ-IR Summary Score can be calculated only for SA women, as in the NSA group it did not meet criterion validity to be considered a valid measure [20], so our study focused only on SA women.

Based on the ROC analysis, the PISQ-IR Summary Score cutoff value in our study was identified as 2.68 for distinguishing women with and without sexual dysfunctions, with values >2.68 representing no sexual dysfunctions. Given the rigorous linguistic and cultural validation process of the PISQ-IR, with the methodology performed according to IUGA protocol [18], it is possible that the newly defined cutoff point of 2.68 could be implemented in other language versions of the questionnaire as well. The minimal and maximal values ranged between 1.545 and 3.909. The values differ in case of patients reporting no sexual partner. Although it would be easier to compare results with a transformed sum score with values from 0 to 100, the authors of the questionnaire advocated the use of the nonweighted summary score and provided the appropriate appendix with the formula [20]. At the same time, Constantine et al. suggested using both domain scores and the single summary score simultaneously, to present the effects of the treatment, because it allows for a separate presentation of the changes in different aspects of sexual function [20].

Sensitivity and specificity are measures of quantifying the diagnostic ability or accuracy of the test. Sensitivity is the ability of a test to correctly identify those with the presence of the feature (true-positive rate), whereas test specificity is the ability of the test to correctly identify those with the absence of the feature (true-negative rate). In our study, the FSFI cutoff score adopted as the reference score allowed for the correct classification to a group without sexual disorders, with 90% sensitivity and 71% specificity. Those results, with the AUC of 0.85, validate that as the cutoff point.

## 5. Concluding Message

Defining the cutoff score of PISQ-IR in women with PFD is an important step in the development of the questionnaire. It can be helpful in analyzing study cohorts and identifying women with sexual function impairment. The PISQ-IR Summary Score below 2.68 represents sexual dysfunction in sexually active women with PFD.

## Figures and Tables

**Table 1:** Female Sexual Function Index domains and scoring [9].

Domain	Questions	Minimum Score	Maximum Score
Desire	1, 2	1.2	6.0
Arousal	3, 4, 5, 6	0	6.0
Lubrication	7, 8, 9, 10	0	6.0
Orgasm	11, 12, 13	0	6.0
Satisfaction	14, 15, 16	0.8	6.0
Pain	17, 18, 19	0	6.0

**Table 2:** Pelvic Organ Prolapse/Incontinence Sexual Questionnaire—IUGA Revised (PISQ-IR) domains and scoring with mean calculation score in sexually active women [17].

Sexually Active (SA) Domains	SA–21 Items	Questions	Minimum Score	Maximum Score
Arousal Orgasm	SA-AO, 4 items	Q 7, 8a, 10, 11	1	5
Condition-specific	SA-CS, 3 items	Q 8b, 8c, 9	1	5
Partner-related	SA-PR, 3 items	Q 13, 14a,14b	1/− *	4/− *
Desire	SA-D, 3 items	Q 15, 16, 17	1	5
Condition Impact	SA-CI, 4 items	Q 18, 20b, 20c, 20d	1	4
Global Quality	SA-GQ, 4 items	Q 19a, 19b, 19c, 20a	1	4.8
Summary Score			1.545	3.909
			1.722 **	3.888 **

* not calculated if no sexual partners are reported by the subject, ** values for the subjects reporting no sexual partner.

**Table 3:** Characteristics of the study population.

Variable	FSFI < 26.55 (*n* = 143)	FSFI > 26.55 (*n* = 83)	*p*-Value
Age (years), mean ± SD	56.8 ± 10.8	56.3 ± 10.6	0.72 ^a^
BMI (kg/m^2^), mean ± SD	27.8 ± 4.6	26.7 ± 4.0	0.06 ^a^
Postmenopausal *n* (%)	107 (74.8)	50 (60.2)	0.02 ^b^
Education *n* (%)			0.09 ^b^
Primary	9 (6.3)	1 (1.2)	
Secondary	79 (55.2)	41 (49.4)	
Higher	55 (38.5)	41 (49.4)	
Parity, mean ± SD (median)	2.4 ± 1.0 (2)	2.1 ± 0.8 (2)	0.02 ^a^
Vaginal delivery	131 (91.6)	69 (83.1)	0.09 ^b^
Cesarean delivery	2 (1.4)	4 (4.8)	
Both	10 (7.0)	8 (9.6)	
None	−	2 (2.4)	
Marital status *n* (%)			0.54 ^b^
married	131 (91.6)	74 (89.2)	
partnership	12 (8.4)	9 (10.8)	
Previous surgical history			
None	93 (65.0)	58 (69.9)	0.47 ^b^
Hysterectomy	29 (20.3)	9 (10.8)	0.08 ^b^
Prior prolapse surgery	35 (24.5)	17 (20.5)	0.49 ^b^
Prior anti-UI surgery	7 (4.9)	5 (6.0)	0.71 ^b^
Clinical diagnosis *n* (%)			
Only UI	34 (23.8)	19 (22.9)	0.62 ^b^
Only POP	56 (39.2)	32 (38.5)	
UI and POP	53 (37.1)	31 (37.3)	
FI	18 (12.6)	5 (6.0)	0.11 ^b^
POP-Q *n* (%)			0.08 ^b^
0	2 (1.4)	6 (7.2)	
I	7 (4.9)	7 (8.4)	
II	52 (36.4)	21 (25.3)	
III	68 (47.6)	42 (50.6)	
IV	14 (9.8)	7 (8.4)	

^a^ Mann-Whitney U test, ^b^ Chi-square test. FSFI, Female Sexual Function Index; BMI, body mass index; UI, urinary incontinence; POP, pelvic organ prolapse; FI, fecal incontinence; POP-Q, pelvic organ prolapse quantification.

**Table 4:** Results of the questionnaires (PISQ-IR and FSFI) in the study groups.

Variable	FSFI < 26.55 (*n* = 143)	FSFI > 26.55 (*n* = 83)	*p*-Value
**PISQ-IR SA** Mean ± SD			
Arousal Orgasm	2.77 ± 0.65	3.71 ± 0.54	0.000 ^a^
Condition-specific	4.11 ± 0.97	4.38 ± 0.70	0.017 ^a^
Partner-related	3.21 ± 0.50	3.50 ± 0.41	0.000 ^a^
Desire	2.70 ± 0.68	3.29 ± 0.56	0.000 ^a^
Condition Impact	2.45 ± 0.78	2.99 ± 0.77	0.000 ^a^
Global Quality	2.78 ± 0.90	3.64 ± 1.01	0.000 ^a^
PISQ-IR Summary Score	2.55 ± 0.38	3.06 ± 0.31	0.000^a^
**FSFI** Mean ± SD			
Desire	2.76 ± 0.91	3.84 ± 0.87	0.000 ^a^
Arousal	3.09 ± 0.96	4.74 ± 0.72	0.000 ^a^
Lubrication	3.56 ± 1.10	5.13 ± 0.80	0.000 ^a^
Orgasm	3.33 ± 1.23	5.13 ± 0.70	0.000 ^a^
Satisfaction	3.98 ± 1.06	5.30 ± 0.79	0.000 ^a^
Pain	3.94 ± 1.34	5.44 ± 0.69	0.000 ^a^
Total Score	20.65 ± 4.34	29.58 ± 2.01	0.000 ^a^

^a^ Level of significance for ANOVA.
